# Ceramic-on-ceramic versus ceramic-on-polyethylene in total hip arthroplasty: a comparative study at a minimum of 13 years follow-up

**DOI:** 10.1186/s12891-021-04950-x

**Published:** 2022-01-17

**Authors:** Malerba Giuseppe, Basilico Mattia, Bonfiglio Nadia, Vitiello Raffaele, Ruberto Pasquale, D’ Adamio Stefano, Sirgiovanni Mattia, De Santis Vincenzo, Maccauro Giulio

**Affiliations:** 1 Fondazione Policlinico Universitario Agostino Gemelli – IRCCS, Largo Agostino Gemelli 8, 00168 Rome, Italy; 2 Università Cattolica Del Sacro Cuore, Roma, Italy; 3 Department of Medical Oncology and Pneumology, University Hospital Tuebingen, Tuebingen, Germany; 4 Cluster of Excellence iFIT (EXC 2180) “Image-Guided and Functionally Instructed Tumor Therapies”, University of Tuebingen, Tuebingen, Germany

**Keywords:** Hip, Replacement, Ceramic, Polyethylene, Wear, outcomes

## Abstract

**Background:**

Nowadays hip replacement is one of the most successful surgery in terms of clinical outcome and patient’s satisfaction. Therefore, the choice of biomaterials in hip replacement is increasingly important with the aim of obtaining a long-term satisfaction of patient and a greater survivorship of the implants. Ceramic-on-polyethylene (COP) and ceramic-on-ceramic (CoC) bearings are two common coupling used in total hip arthroplasty. The aim of this retrospective study was to compare clinical and radiological outcomes between patients treated using CoC and CoP THA at a mean follow-up of 15 years.

**Methods:**

86 patients, average age 65.6, were included in the study: 43 in group A bearing CoC and 43 in group B bearing CoP. Minimum follow-up was 13 years.

Primary outcome was a clinical evaluation assessed by HOOS and SF-12 questionnaires.

Secondary outcome was a radiological evaluation on a A-P pelvis x-ray calculating acetabular cup inclination and anteversion and detecting osteolysis.

**Result:**

After a multivariate analysis was performed, our results show clinical outcomes in group B significantly better than in group A: statistically significant value (*p* < 0,05) was found in the mean HOOS-symptoms subscale (83.0 ± 15.4 in Group A vs 90.3 ± 12.2 in group B) in the SF-12 physical component score (39.7 ± 11.0 in Group A vs 48.1 ± 10.1 in group B) and in HOOS (79.0 ± 16 in Group A vs 87.0 ± 16 in group B). 3 squeaking was found in group A. The calculated mean acetabular cup inclination value was 44,87 in group A and 44,5 in group B and the mean socket version was 17,54 in group A and 15,10 in group B. No significant statistically relationship between radiographic parameters analyzed and clinical outcomes was noted.

**Conclusion:**

The current results provide us important information about the THA long-term outcome. CoP offered significantly better results compared with CoC at long-term follow up, and thus it should be considered in the choose of bearing in THA.

## Background

Total hip arthroplasty (THA) is an increasingly frequent treatment nowadays, and further increase in use of THA is expected, so as its optimal outcome [1].

Classically THA surgery was reserved for elderly patients. In recent years, however, there has been a reduction in the average age of patients undergoing THA and very young patients are subjected to it due to post-traumatic osteoarthritis, osteonecrosis, inflammatory arthritis, and congenital deformities (dysplasia of the hip, Legg-Calvè-Perthes disease, slipped capital femoral epiphysis) [2, 3].

For this reason, the physical demands of the patients are increased, and more attention has been given to the biomaterials of the implants and their combinations, with the aim of obtaining a longer functionality of the implants.

Aseptic loosening following wear debris is classically considered the main cause of long-term failure after total hip arthroplasty [4].

Revision surgery is technically complex, it is linked to high risk of complications, morbidity and poor clinical outcome with the consequent economical burden on the healthcare system [5].

In this regard, the biomechanical studies are oriented in an attempt to minimize the wear between the various components, between the neo-femoral head and the acetabular liner.

Materials used for this purpose encompass metal, polyethylene, and bioceramics.

Combinations include metal-on-polyethylene, metal-on-metal, ceramic-on-ceramic (CoC), ceramic-on-polyethylene (CoP) and ceramic heads and metallic inserts.

Several studies have focused on the materials in hip arthroplasty: the ceramic-ceramic combination seems to better withstand mechanical wear and a longer implants life is expected [6, 7].

In this regard, it is important to compare the clinical outcomes and the satisfaction of patients undergoing total hip replacement (THR) using different materials and combinations.

The aim of this retrospective study is to compare the clinical and radiological outcome of patients treated using CoC and CoP at a minimum 13-years follow-up.

## Methods

An integrated hospital system total joint replacement database was used to identify a cohort of patients with primary elective THAs performed in our hospital from January 1, 2005, to December 31, 2008. 103 subjects undergoing THA by the same surgeon in our hospital between 2005 and 2008 were recruited. Inclusion criteria were: patients undergoing primary THA from 2005 to 2008 performed by one senior surgeon; expression of informed consent to take part in the study; the same stem (Accolade-Stryker® Kalamazoo, Michigan, USA) and acetabular cup used for all subject (Trident-Stryker® Kalamazoo, Michigan, USA); patients aged > 18 years.

Exclusion criteria were: patients undergoing revision hip arthroplasty due to mechanical complications, infectious complications or aseptic loosening.

As a primary outcome was chosen the comparison of clinical assessment between CoC vs CoP THAs.

Secondary outcome was radiographic evaluation on antero-posterior pelvis x-ray.

In January 2021 subjects were contacted by telephone to confirm vital status and to schedule an outpatient visit.

The patients in group A received a ceramic head 2005 (BIOLOX® Forte; CeramTec AG, Plochingen, Germany) coupled with a ceramic insert (BIOLOX-forte, CeramTec, Plochingen, Germany).

The patients in group B received a ceramic head 2005 (BIOLOX® Forte; CeramTec AG, Plochingen, Germany) coupled with a highly cross-linked polyethylene insert (Stryker Orthopaedics Mahwah, New Jersey, USA).

These bearing surfaces were chosen because already in the early 2000s they were considered the most interesting and potentially the best performing in terms of wear resistance and survival tought the exact ‘best’ option is nowdays unknown [8, 9].

All THAs were performed with the patient in the lateral position, using the Gibson-Moore posterolateral approach [10].

### Post-surgery routine

No intra-articular drainage was positioned after surgery [11]; postoperatively, the patient’s pain was treated with paracetamol, NSAIDs or Oxycodone as needed.

Patients were weightbearing as tolerated on the leg involved.

One day postoperative occupational and physical therapy were initiated, and walking was allowed with crutches. No antibiotic was used in post-surgery protocol.

Antithrombotic prophylaxis consisted in the use of graduated compression stockings and low-molecular-weight Heparin once daily for 5 weeks post-surgery.

### Clinical evaluation

After their expression of consent, during an external consultation the patients were clinically evaluated for restoring range of motion, presence of hip pain and evocable audible noise of the hip as squeaking, clicking, grinding.

Squeaking has been considered as a high-pitched audible sound from the hip; clicking as a “click” that occurs during hip movement or walking; grinding as “crepitus” during movement [12].

At the moment of the visit the subjects completed two questionnaires: HOOS (Hip disability and Osteoarthritis Outcome Score) e SF12 (12-Item Short Form Survey).

The HOOS is a 40-item self-reported questionnaire comprising 5 subsets: pain, symptoms, activities of daily living, sport and hip related quality of life. The score is expressed as a percentage with a higher value corresponding to a higher patient satisfaction [13, 14].

The SF12 is a self-reported outcome measure assessing the impact of health on an individual’s everyday life, being a shortened version of the SF-36 and it is often used as a quality-of-life measure [15, 16].

Total scores allow to construct two synthetic indices: a physical health index (SF-12 P) and a mental health index (SF-12 M). The lower the score of the two indices, the greater the level of disability.

### Radiological evaluation

During the outpatient consultation, an antero-posterior pelvis x-ray was obtained, and a radiographic evaluation was performed to calculate hip socket inclination and anteversion.

**Fig. 1:**
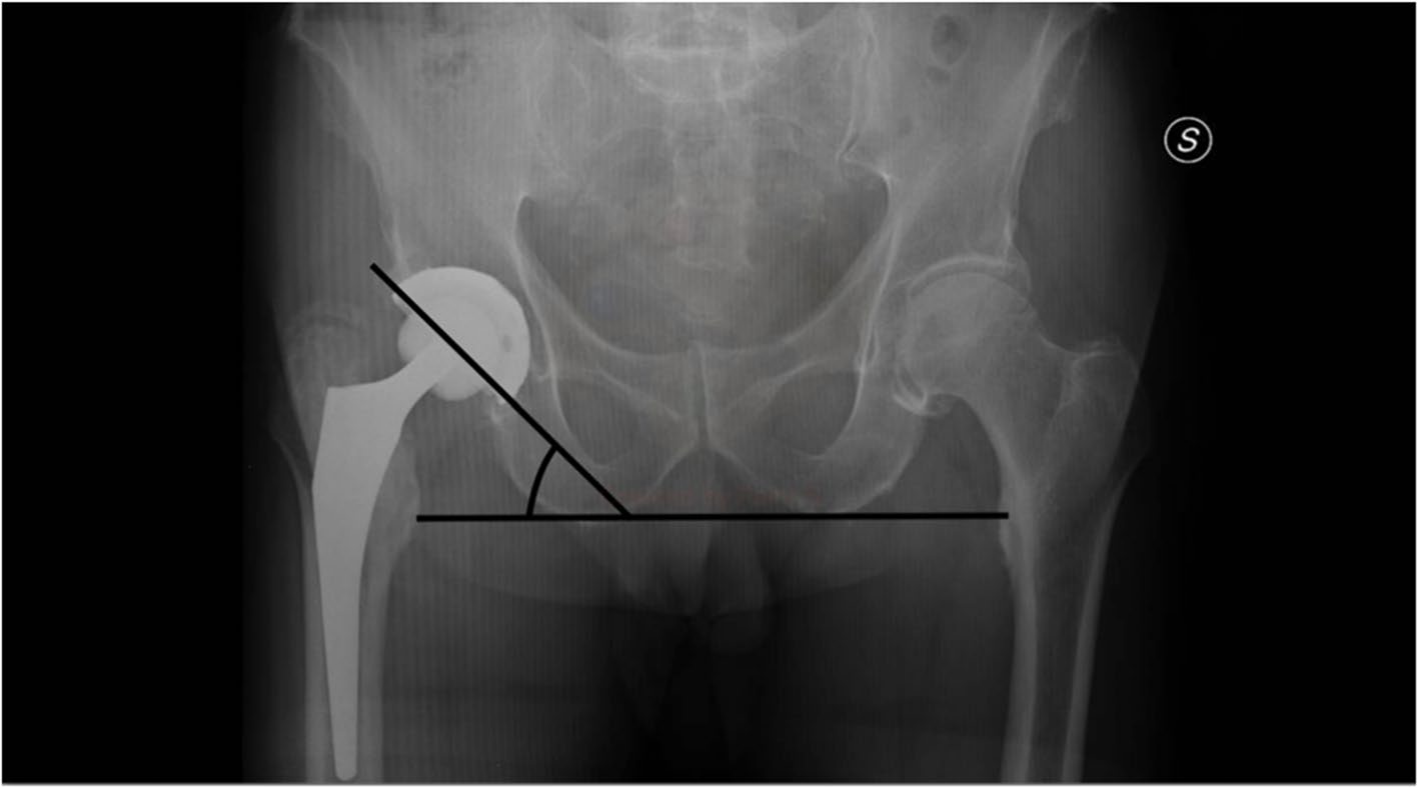
Acetabular cup inclination. Method of calculation of acetabular cup inclination on AP pelvic radiographs as the angle formed between a line drawn along the opening of the acetabular component and one joining the ischial tuberosities

The inclination was measured on standard X-ray as the angle between a line drawn along the opening of the acetabular component and one joining the ischial tuberosities [17] (Fig.1).

**Fig. 2:**
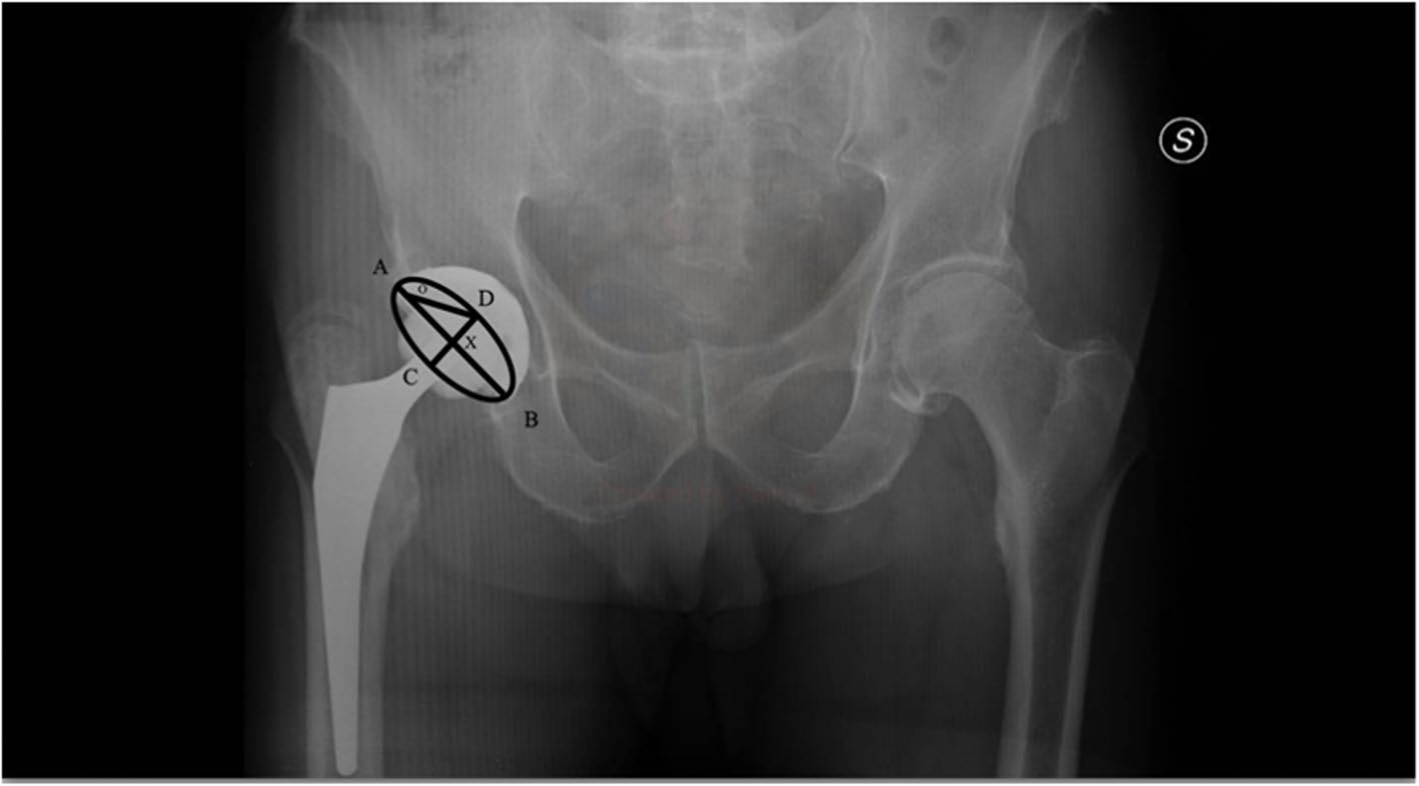
Acetabular cup ante-version. Calculation of acetabular cup anteversion on AP pelvis radiograph using method explained by V. Bachhal et al. AB = major axis of the ellipse, CD = minor axis of the ellipse represented acetabular component XOD = the calculated angle of anteversion

Planar anteversion is the rotation of the acetabular face along the axis defined by the intersection of the coronal plane and the plane of the acetabular face. It is calculated on plane x-ray following the method explained by V. Bachhal et al. which proved to be simple to perform and reliable [18] (Fig. 2).

**Fig. 3:**
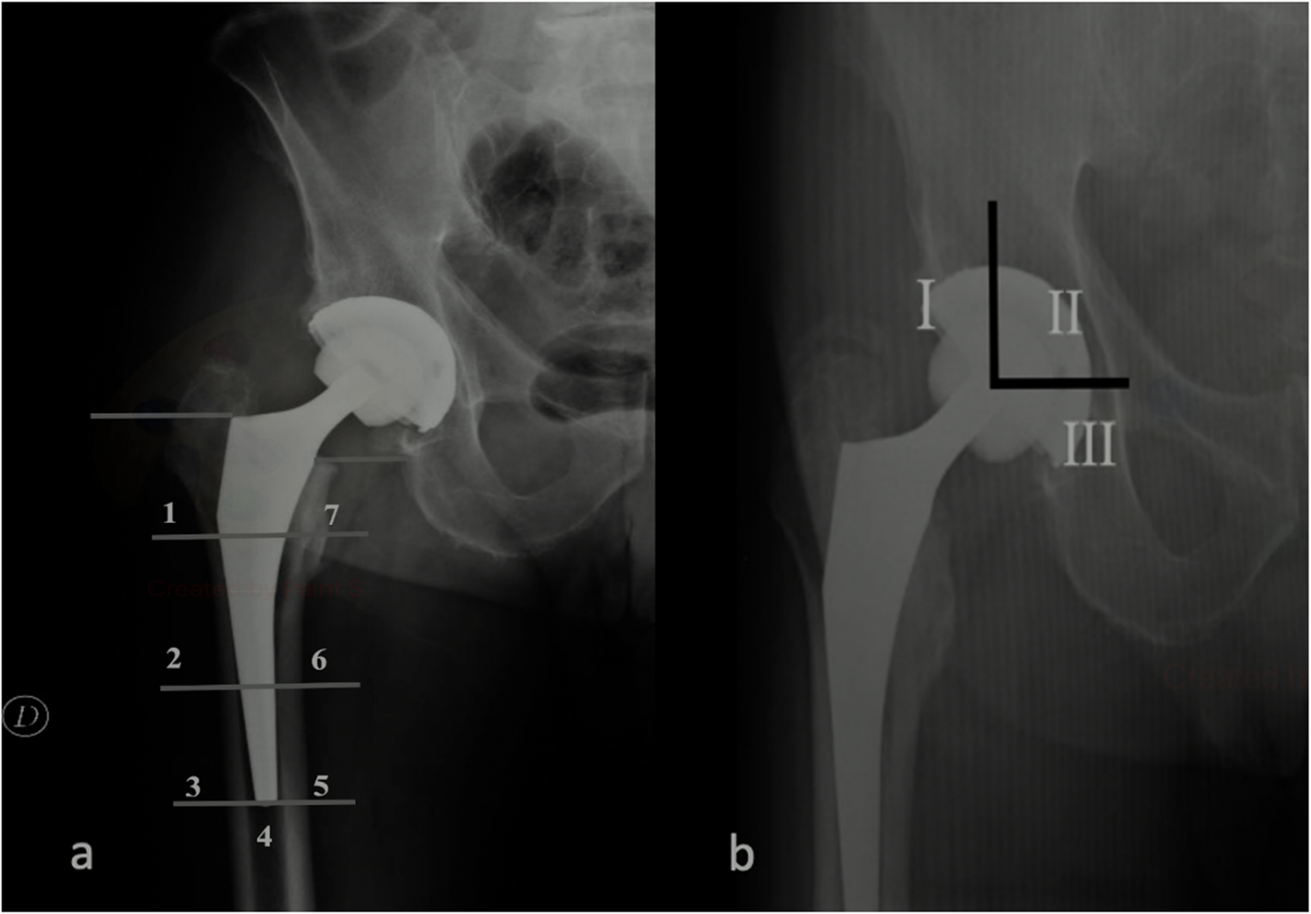
Areas of osteolysis. 7 areas of femoral osteolysis according to Gruen zones (**a**) and 3 areas of peri-acetabular osteolysis according to De Lee and Charnley zones (**b**)

Finally, areas of osteolysis were reported on plane radiographs according to (A) Gruen zones and (B) De Lee and Charnley zones. (Fig. 3). Osteolysis was defined as the appearance of a radiolucid zone in the pelvic area around the acetabulum and in the femoral shaft near the femoral stem [19] (Fig. 3).

### Statistical analyses

STATA 16 was used for statistical analyses. We considered *P* values < 0.05 to be significant.

Since the variables we investigated were continuous scales we first examined them by mean of T-Test and then we added adjusting factors performing a multiple linear regression analysis. Adjusting factors were chosen in order to keep low the risk of overfitting the model, while maintaining in it factors known to affect the outcome.

## Results

A total of 103 Total Hip Arthroplasties were enlisted in our study.

17 (16,5%) were excluded: 10 patients (11%) due to loss of follow-up and 7 patients (6,5%) were revised due to THA dislocation (2 hips), aseptic loosening (3 hips) and periprosthetic fracture (2 hips)

**Fig. 4:**
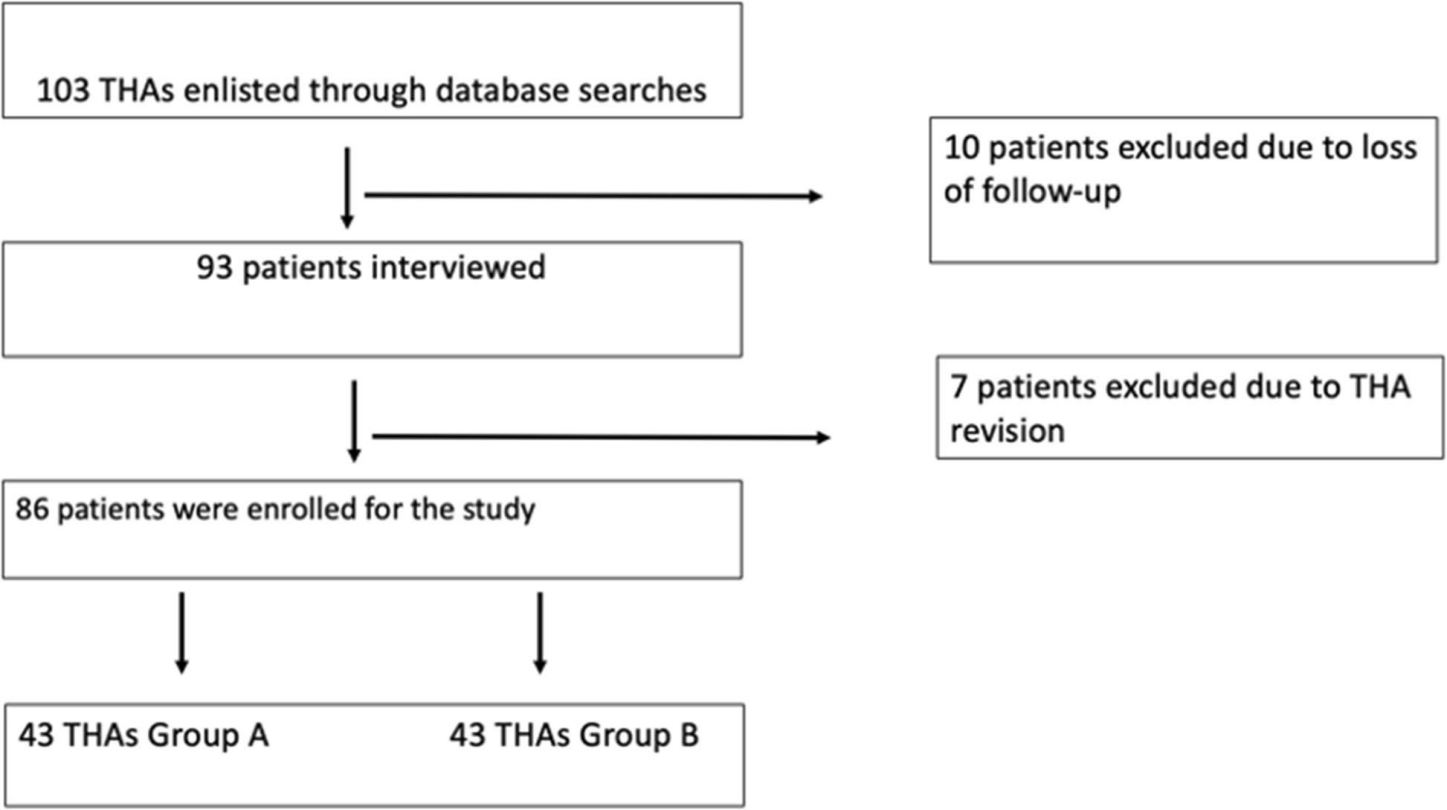
Inclusion process of patients. Flowchart summarizing the selection process of the subjects in the study

Finally, 86 patients were enrolled in the study, divided into two groups: group A CoC (43 subjects) consisting of patients with the ceramic-ceramic bearings and group B CoP (43 subjects) with ceramic-polyethylene bearings (Fig. 4).

In group A there were 20 female and 23 males, in group B there were 22 female and 21 males.

Average age of the patients was 65.6 years, with a mean of 63.4 years for group A (SD 6.5) and 67.8 years for group B (SD 11).

The average BMI was 25.9 (SD 3.3) for the group B and 27 (SD3.1) for the group A.

The mean follow-up was 15 years (SD 1) with a minimum of 13 years.

All parameters showed an asymmetry between − 2 and + 2, suggestive of a normal distribution.

The adjusted results, for age, BMI and gender, showed no significant difference between the two groups regarding HOOS-P, HOOS-A, HOOS-Sp, HOOS-Q, SF12p and SF12M once the analyses were corrected for the presence of the aforementioned factors (Table 1).

**Table 1:** Clinical outcomes

	Group A (CoC)	Group B (CoP)	p	95% CI
Hoos – S	83.0 (SD 15.4)	90.3 (SD 12.1)	0.04*	0.33 to 15.59
HOOS – P	85.5 (SD 11.8)	91.4 (SD12.9)	0.052	−0.04 to 13.06
HOOS – SP	79.4 (SD 23.7)	79.4 (SD 23.7)	0.08	−1.51 to 24.03
HOOS – A	77.9 (SD 17.7)	86.8 (SD 17.7)	0.06	−0.46 to 20.52
HOOS – TOTAL	79 (SD 16)	87 (SD 16)	0.04*	0.24 to 17.90
SF-12 P	39.7 (SD 11.0)	48.1 (SD 10.1)	0.02*	3.60 to 15.03
SF-12 M	46.1 (SD 11.3)	49.8 (SD 8.6)	0.14	−1.13 to 9.97

Data were reported as absolute value and in brackets SD. The * underline statistical significance

The HOOS-S Score however diverged significantly from this trend, being significantly higher in the group B than in the Group A (CI: 0.33 to 15.59, *p* = 0.04).

A subsequent analysis also combined the scores to obtain a so called “HOOS total”. According to these analyses the group B showed a score of 9.07 points higher than the Group A (CI: 0.24 to 17.90, p = 0.04). 4 patients in the group A (3/43 patients, 6%) reported occasionally hearing sounds from their THA: the reported sound is like a “squeak”, no such anomaly was reported in group B.

In group A the calculated mean socket inclination value was 44.87 and mean socket version was 17.54. In group B the mean inclination was 44.5 and the mean version was 16.10. Pearson Correlation Coefficient was used to analyze relationship between anteversion cup and clinical outcomes, no statistical difference was noted.

Finally, we identified one osteolysis in zone 1 according with Gruen zone in group A (1/43 patients, 2%) and two osteolysis (1 in zone 1 according with De Lee and Charnley classification and 1 in zone 7 according with Gruen zones) in group B (2/43 patients, 4%), no statistical difference was noted (*p* = 0.5).

## Discussion

Long-term patient satisfaction and implant survival depends on their wear resistance and it is associated with the particulate debris´ release because of wearing [20].

In hip prosthetics, the study of tribology has always been a debated topic. Our study is our contribution on the debate about materials: comparing functional outcome and satisfaction between CoC and CoP after THR at minimum 13 years f-u, our results show a statistically significant difference between the two groups about clinical outcome in favor of CoP group versus CoC group.

Ceramic on ceramic bearing were presented as a solution to wear, given the good wear resistance of ceramic components [21–23].

Several authors proved this coupling allows to achieve good medium-long term outcome, especially in young patients: Solarino et al. reported excellent results in 200 Ceramic-on-Ceramic cementless hip arthroplasties in young patients rated 5–24 years follow-up after surgery [24–28].

Likewise, analyzing 113 primary THAs in 91 patients younger than 20 years at the time of surgery, Hannouche et al. reported a satisfactory outcome at 8.8 years-medium follow up: the mean HOOS score was 79.3 ± 13.8 [29].

Our study also showed a good long-term outcome with a HOOS mean value of 79 (SD 16) in our sample patient treated using CoC bearing, despite a higher average age.

However, CoC bearing is related to important drawbacks: potential for breakage and noisy after hip movement.

Actually, the breakage of components is not a common occurrence: according to a recent meta-analysis the rate of ceramic fracture was 0.9/1000 patient-year in THA using Forte ceramic and 0.5/1000 patient-year using Delta ceramic [30].

Liner fracture is reported in literature with a higher frequency than head, being between 0.13 to 1.1% [31, 32].

Furthermore, the introduction of the latest generations of ceramic seems to have solved the problem of head fracture and greatly reduced problem of the liner breakage. Indeed, our study, analyzing ceramics from 2005 to 2008, did reveal no breakage of component in the CoC sample under examination.

Another problem related to CoC bearing consists of unwanted sounds from the hip after THA. The main reported noise is the squeaking. Although it does not affect the functional outcome of the THA, it plays an important role in patient satisfaction [33].

In some cases the squeaking was so unbearable that hip-replacemente was required, despite the full functionality to the hip [34].. There is no agreement about the real rates of ceramic-on-ceramic squeaking, although it seems to fluctuate between 1 and 20.9% [35, 36].

We found three (6% of CoC group) squeakings in CoC group, but no patient with “noisy hip” required a re-operation because of that.

Yet, with ceramic- on polyethylene bearing being a good alternative to CoC, the choice of the best materal coupling is still a matter of debate.

However, the use of polyethylene, especially old generation polyethylene, is burdened by a greater accumulation of wear debris and consequent osteolysis [37].

We investigated the presence of osteolysis on planar x-rays and identified one osteolysis in group A and two in group B. After an analysis they did not seem to influence the patient functional outcome in our groups.

After a multivariate analysis was performed, our results showed the degree of satisfaction of patients in group B being significantly better than in group A, measured by the HOOS and SF-12 questionnaire.

This result differs from many studies comparing the outcomes of CoC and CoP in THA that showed no significant differences between the two groups in terms of pain, stiffness, patient satisfaction, component wear, and failure or revision rate [38–43].

These data explain why the choice of materials in THA is still matter of debate nowadays and the consensus on material components has not yet been reached.

Furthermore, the use of the polyethylene liner has an important economic value, resulting in savings compared to the higher cost of the ceramic [44].

### Radiographic evaluation

The correct orientation of the acetabular cup is essential for a good outcome after a total hip arthroplasty.

Lewinnek has identified a safe zone of anteversion of 15° +/− 10° and inclination of 40° +/− 10° in which the dislocation rate is lower than outside this range [45, 46].

Grammatopoulos et al., demonstrated the best outcomes were achieved with an inclination of 45° ± 5° and an anteversion of 25° ± 5°(Δ Oxford Hip Score.

> 26), after analyzing the orientation of acetabular component in 1070 primary THR with hard-on-soft [47].

Many authors tried to identify the correct orientation of the cup, but even more have tried to devise methods to obtain the correct and desired orientation of the cup intraoperatively [48–50].

The orientation of the sockets analyzed in our study fall within the Lewinnek safe zone, and no significant correlation was found between socket orientation and clinical outcome.

### Limits

The results of this study must be considered in light of its retrospective nature and its small sample size, being its major limitations. Because of that, no preoperative evaluation could be obtained to correlate the pre- and post-operative outcomes, thus including underlying diseases influencing the parameters considered.

For the same reason, the long-term functionality of the implant was not examined, but the main focus was put on the evaluation of the degree of patient satisfaction in relation to material components years after surgery.

Despite these weaknesses, some strengths of the study need to be underscored, among which are surely the long follow-up and the uniformity of the sample. To the current authors’ knowledge, in the literature few studies compare bearings in hip replacements at such a long follow-up.

Moreover, we tried to limit the bias by including THAs performed by the same surgeon using the same stem and the same acetabular cup.

This paper receives funding of Orthopedic and Traumatology School of Università Cattolica del Sacro Cuore – Roma. The funders did not play any role in the design of the study, the collection, analysis, and interpretation of data, or in writing of the manuscript.

## Conclusion

Biomaterial remains a debated topic for Hip Arthroplasty. Despite that, the current results provide us important information about the long-term good outcome of CoP bearing compared to CoC’s ones. In fact, it is suggested that the low wear clinical performance related to ceramic-on-ceramic bearings can be challenged by the performance of highly cross-linked polyethylene, also thanks to the reliability of the new polyethylene which seems to have overcome the wear problems related to the first generation of polyethylene. In view of the results, the bearing surface Ce-Po seems to be more resistant to wear than previously thought and could be considered as an option even in young patients with high functional demand.

However, further prospective studies would be needed, with a larger sample, to obtain more complete information about the bearing surface and the THAs survivorship.

## Data Availability

The datasets used and/or analyzed during the current study are available from the corresponding author on reasonable request.

## Abbreviations

THA: Total hip replacement
CoC: Ceramic-on-ceramic
CoP: Ceramic-on-polyethylene
HOOS: Hip disability and Osteoarthritis Outcome Score
SF12: 12-Item Short Form Survey
